# Midline Shift is Unrelated to Subjective Pupillary Reactivity Assessment on Admission in Moderate and Severe Traumatic Brain Injury

**DOI:** 10.1007/s12028-018-0526-8

**Published:** 2018-04-04

**Authors:** Basil Nourallah, David K. Menon, Frederick A. Zeiler

**Affiliations:** 1 Division of Anaesthesia, Department of Medicine, Addenbrooke’s Hospital, University of Cambridge, Cambridge, UK; 2 Department of Surgery, Rady Faculty of Health Sciences, University of Manitoba, Winnipeg, MB R3A 1R9 Canada; 3 Clinician Investigator Program, Rady Faculty of Health Sciences, University of Manitoba, Winnipeg, Canada

**Keywords:** Traumatic brain injury, Midline shift, Basal cisterns, Pupillary reactivity

## Abstract

**Background:**

This study aims to determine the relationship between pupillary reactivity, midline shift and basal cistern effacement on brain computed tomography (CT) in moderate-to-severe traumatic brain injury (TBI). All are important diagnostic and prognostic measures, but their relationship is unclear.

**Methods:**

A total of 204 patients with moderate-to-severe TBI, documented pupillary reactivity, and archived neuroimaging were included. Extent of midline shift and basal cistern effacement were extracted from admission brain CT. Mean midline shift was calculated for each ordinal category of pupillary reactivity and basal cistern effacement. Sequential Chi-square analysis was used to calculate a threshold midline shift for pupillary abnormalities and basal cistern effacement. Univariable and multiple logistic regression analyses were performed.

**Results:**

Pupils were bilaterally reactive in 163 patients, unilaterally reactive in 24, and bilaterally unreactive in 17, with mean midline shift (mm) of 1.96, 3.75, and 2.56, respectively (*p* = 0.14). Basal cisterns were normal in 118 patients, compressed in 45, and absent in 41, with mean midline shift (mm) of 0.64, 2.97, and 5.93, respectively (*p* < 0.001). Sequential Chi-square analysis identified a threshold for abnormal pupils at a midline shift of 7–7.25 mm (*p* = 0.032), compressed basal cisterns at 2 mm (*p* < 0.001), and completely effaced basal cisterns at 7.5 mm (*p* < 0.001). Logistic regression revealed no association between midline shift and pupillary reactivity. With effaced basal cisterns, the odds ratio for normal pupils was 0.22 (95% CI 0.08–0.56; *p* = 0.0016) and for at least one unreactive pupil was 0.061 (95% CI 0.012–0.24; *p* < 0.001). Basal cistern effacement strongly predicted midline shift (OR 1.27; 95% CI 1.17–1.40; *p* < 0.001).

**Conclusions:**

Basal cistern effacement alone is associated with pupillary reactivity and is closely associated with midline shift. It may represent a uniquely useful neuroimaging marker to guide intervention in traumatic brain injury.

## Introduction

Severe traumatic brain injury (TBI) is a major cause of mortality and morbidity. Patients with TBI require rapid neurological examination, including assessment of pupil reactivity to light and consciousness level, and diagnostic evaluation by brain computed tomography (CT). Both pupil reactivity and CT characteristics provide important information to guide definitive management in TBI. Furthermore, they are main baseline variables included within the two main prognostic models quoted in the TBI literature, the CRASH and IMPACT models [1–5].

Early anatomic studies demonstrated that pupillary dilatation commonly results from raised intracranial pressure (ICP) causing oculomotor nerve entrapment due to uncal herniation across the tentorial incisura, signaling ongoing or impending mechanical compression of the brainstem [6–8]. Prognostic scoring systems for brain CT in TBI emphasize imaging stigmata of raised ICP, such as midline shift and basal cistern effacement [9–12], and among clinicians, there is a view that an unreactive pupil can predict a degree of supra-tentorial mass effect. However, the relationship between pupillary reactivity and these imaging characteristics in TBI has not been well described. Moreover, the nature and degree of the relationship between the different imaging features themselves is unclear.

Thus, elucidating the relationships between midline shift, basal cistern effacement and pupillary reactivity is important for understanding both the evolution of supra-tentorial mass effect on CT and the precise diagnostic utility of an unreactive pupil in the brain-injured patient. These relationships have not been previously explored in depth. One study of 245 patients found that patients with bilaterally unreactive pupils who scored 3 on the Glasgow Coma Scale (GCS) were more likely to have midline shift > 5 mm present on their CT scan [13], while a previous small case series of 15 patients found no such association [14]. The purpose of the present study is to systematically investigate these relationships.

## Methods

### Study Design

This is a cohort study with retrospective analysis of available patient data within electronic patient records and archived neuroimaging. From a database of 358 patients with TBI, we identified 204 patients for whom both CT imaging and pupil reactivity status were available. All patients were admitted to the Neurosciences and Trauma Critical Care Unit (NCCU) at Cambridge University Hospitals NHS Foundation Trust between March 2005 and December 2016. Patients suffered either moderate-to-severe TBI or mild TBI and subsequently deteriorated to a point where they required ICP monitoring, sedation and mechanical ventilation as part of ICP management. Demographic data (sex, age, admission GCS and pupillary response) were recorded and therefore available to the study retrospectively through database analysis. Since all data were extracted from the hospital records and fully anonymized, no data on patient identifiers were available, and formal patient or proxy consent was not required.

The first assessment of pupil reactivity status recorded in the emergency department and noted in the archived electronic medical records was extracted and recorded as normal bilaterally, unilateral unreactive, or bilaterally unreactive in accordance with the IMPACT model criteria [1, 3]. The first admission CT was used for each patient to evaluate midline shift and basal cisterns status. The exact time between assessment of pupillary reactivity and CT is unknown, but likely within 1–2 h in most cases. This time period was selected to avoid confounding by sedation, as subsequent CTs were more likely to be conducted under sedatives, and progression of intracranial injury after admission clinical assessment. CT images were evaluated by a single consultant neurosurgeon blinded to pupillary status to determine extent of midline shift, lesion type, and basal cistern appearance. Midline shift was assessed on admission brain CT, using the distance of the septum pellucidum from bony midline (derived from the line connecting the crista galli to the inion) at the level of the foramen of Monro. Basal cisterns were graded via an ordinal system (0 = normal, 1 = compressed, 2 = absent/completely effaced).

### Statistical Analysis

Descriptive statistics were applied to summarize demographic data. Pupil reactivity was expressed as an ordinal variable in accordance with the IMPACT classification of bilaterally reactive, unilaterally unreactive, and bilaterally unreactive [3]. Additionally, two binary classifications were used, namely normal versus abnormal (at least one unreactive pupil) and bilaterally unreactive versus other (at least unreactive pupil), with the goal of comparing normal against any abnormality, and extreme neurological impairment against other. Basal cistern appearance was recorded as an ordinal variable with levels normal, compressed, and absent. Two binary classifications were also used, namely normal versus any compression, and absent versus not completely effaced. The mean midline shift of patients in different pupil reactivity categories was calculated, and statistical significance was assessed by the Kruskal–Wallis test, Mann–Whitney *U* test, and Jonckheere–Terpstra test as appropriate. This analysis was repeated for subsets of patients with focal and diffuse injury. Sequential Pearson Chi-square calculations were performed at 0.25 mm increments of midline shift to determine whether there is a threshold of midline shift which most accurately predicts abnormal pupil reactivity in either binary classification. The same analyses were applied to both ordinal and binary basal cistern status categorizations. Additionally, in order to assess the possible impact of selection bias on results, nonparametric statistical tests of independence were used to compare average age, sex, GCS, midline shift, and basal cistern status in patients with and without available admission pupillary reactivity data.

Univariable logistic regression analysis was performed with midline shift as a predictor variable to derive the odds ratio for the aforementioned binary classifications of pupil reactivity and basal cistern status. Multiple logistic regression analysis was subsequently performed with midline shift, basal cistern effacement, and age included as predictors as pupillary reactivity. Again, this analysis was repeated for subsets of patients with focal and diffuse injury. All statistical analysis was performed using R statistical software (R Core Team (2016). R: A language and environment for statistical computing. R Foundation for Statistical Computing, Vienna, Austria. URL https://www.R-project.org/) with statistical significance set at *p* < 0.05. Graphical production was completed using the ggplot2 package in R.

## Results

### Cohort Characteristics

A total of 204 patients had documented pupil reactivity on admission accessible on electronic records and were included the study: 161 males and 43 females with a median age of 44 (range 16–89). Forty-one patients had one or two unreactive pupils on admission, while 86 had abnormal basal cistern appearance on CT. In 122 patients, the largest lesion detected on CT was focal, whereas 82 patients had diffuse injury. No patients had CT evidence of contralateral hydrocephalus or Duret hemorrhages. Cohort descriptive statistics are presented in Table 1.

**Table 1:** Cohort descriptive statistics

Measure	Subcategory or units	Total cohort
Median age (IQR)	Years	44 (24.75–56)
Gender	Male/female	161: 43
Mechanism	RTA	94
	Fall	44
	Assault	13
	Other	4
	Unknown	49
Pupil reactivity on admission	Bilaterally reactive	163
	Unilaterally reactive	24
	Bilaterally unreactive	17
Basal cistern appearance on admission CT	Normal	118
	Compressed	45
	Absent	41
Largest lesion type [frequency, mean, and range of midline shift (mm)]	SDH	74, 4.45 (0.0–19.8)
	DAI	43, 0.23 (0.0–6.7)
	Contusion	29, 1.66 (0.0–11.4)
	EDH	17, 3.29 (0.0–14.0)
	Falcine SDH	1, 7.4
	Posterior fossa EDH	1, 0
	No mass lesion detected	39 0.054 (0–2)
Median GCS on admission (IQR)		7 (4–10)
Median ISS (IQR)		33 (25–41)

*CT* computed tomography, *DAI* diffuse axonal injury, *EDH* extradural hematoma, *GCS* Glasgow Coma Scale, *IQR* interquartile range, *ISS* injury severity score, *RTA* road traffic accident, *SDH* subdural hematoma

Age, sex, GCS, and basal cistern status were not significantly different in the patients with and without available admission pupillary reactivity data. However, mean midline shift was significantly increased in patients with available pupillary reactivity data (2.22 vs. 1.15 mm, *p* = 0.023) (Table 2).

**Table 2:** Demographic data by admission pupillary reactivity data availability

	Pupils data available (*n* = 204)	Pupils data not available (*n* = 154)	*p* value
Median age	44	37	0.054
Sex	Male 161 Female 43	Male 111 Female 43	0.17
Median GCS	7	6	0.17
Mean midline shift	2.22 mm	1.15 mm	**0.023**
Basal cistern status	Normal 118 Compressed 45 Absent 41	Normal 100 Compressed 36 Absent 18	0.10

*GCS* Glasgow Coma Scale

Bold *p* values are those reaching statistical significance

#### Mean Midline Shift and Pupil Reactivity

**Fig. 1:**
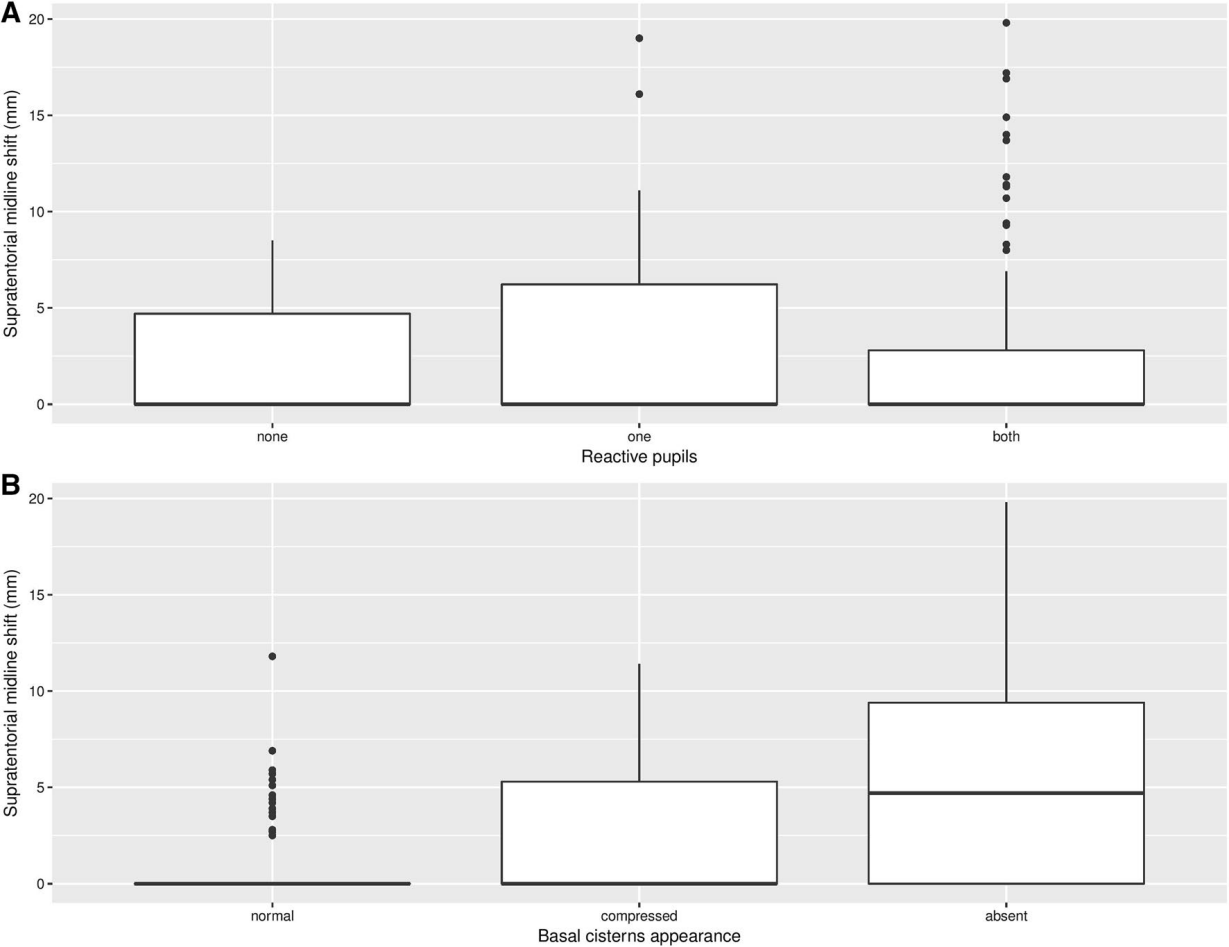
**a** Box plot of supra-tentorial midline shift by pupillary reactivity. **b** Box plot of supra-tentorial midline shift by basal cistern appearance

Pupil reactivity was expressed as an ordinal variable with categories of bilaterally reactive, unilaterally reactive, and bilaterally unreactive. Mean midline shift (mm) was 1.96, 3.75, and 2.56, respectively (*p* = 0.14). To test the hypothesis that midline shift is sequentially increasing among these categories, Jonckheere–Terpstra test was applied (*p* = 0.027). Midline shift data for each category are represented graphically in Fig. 1.

Patient were also assigned to a binary group of normal versus abnormal pupils, giving mean midline shift (mm) of 1.96 and 3.25, respectively (*p* = 0.047). A second binary characterization grouping patients with bilaterally unreactive pupils versus those with at least one reactive pupil produced mean midline shift (mm) of 2.56 versus 2.18 (*p* = 0.22) (Table 3). Separating patients by nature of intracranial injury (focal vs. diffuse) resulted in no significant differences in either group (Tables 4, 5, 6, 7, 8, 9, 10, 11).

**Table 3:** Mean midline shift by pupil reactivity

Pupil responses	Group	Mean MLS (mm)	Variance	*p* value
Normal versus unilaterally reactive versus bilaterally unreactive	All	1.96 versus 3.75 versus 2.56	15.21 versus 32.07 versus 9.96	0.14 (0.024 by JT)
At least unilaterally fixed versus other	**All**	**3.25 versus 1.96**	**22.47 versus 15.21**	**0.047**
Bilaterally fixed versus other	All	2.56 versus 2.18	9.96 versus 17.57	0.22

*JT* Jonckheere–Terpstra test, *MLS* midline shift. Bold *p* values are those reaching statistical significance (*p* < 0.05)

**Table 4:** Mean midline shift by pupil reactivity in focal lesions

Pupil responses	Mean MLS (mm)	Variance	*p* value
Normal versus unilaterally reactive versus bilaterally unreactive	3.25 versus 5.63 versus 3.77	21.57 versus 37.92 versus 11.00	0.22 (0.046 by JT)
At least unilaterally fixed versus other	4.87 versus 3.25	26.97 versus 21.57	0.090
Bilaterally fixed versus other	3.77 versus 3.60	11.00 versus 24.30	0.45

*JT* Jonckheere–Terpstra test, *MLS* midline shift

**Table 5:** Logistic regression results including age and basal cistern appearance in focal lesions

Predictor variable	Univariable regression odds ratio (95% CI; *p* value)		Multiple regression odds ratio (95% CI; *p* value)	
	Normal pupils versus at least unilaterally fixed	At least one pupil reactive versus bilaterally fixed	Normal pupils versus at least unilaterally fixed	At least one pupil reactive versus bilaterally fixed
Basal cisterns normal	Reference	Reference	Reference	Reference
Basal cisterns compressed	0.58 (0.18–1.93; 0.36)	**0.10 (0.0052**–**0.75; 0.048)**	0.58 (0.17–2.00; 0.37)	**0.077 (0.0037**–**0.58; 0.028)**
Basal cisterns absent	**0.22 (0.076**–**0.60; 0.0039)**	**0.072 (0.0037**–**0.45; 0.017)**	**0.20 (0.058**–**0.68, 0.011)**	**0.041 (0.0020**–**0.30;0.0062)**
Midline shift	0.94 (0.86–1.02; 0.13)	0.99 (0.88–1.15; 0.91)	1.01 (0.91–1.12; 0.84)	1.11 (0.97–1.32; 0.17)
Age	0.99 (0.97–1.02; 0.77)	1.00 (0.97–1.04; 0.83)	0.99 (0.96–1.02; 0.63)	1.00 (0.96–1.036; 0.86)

Multiple regression includes basal cistern status, midline shift, and age as predictor variables. Bold *p* values are those reaching statistical significance (*p *< 0.05)

**Table 6:** Mean midline shift by basal cistern status in focal lesions

Basal cistern status	Mean MLS (mm)	Variance	*p* value
Normal versus compressed versus absent	1.24 versus 4.37 versus 7.44	5.72 versus 12.47 versus 40.77	< 0.001 (0.001 by JT)
Normal versus any compression	1.24 versus 5.98	5.72 versus 29.27	< 0.001
Visible versus absent	2.25 versus 7.44	9.94 versus 40.77	< 0.001

*JT* Jonckheere–Terpstra test, *MLS* midline shift

**Table 7:** Univariable logistic regression results with midline shift as a predictor of basal cistern status in focal lesions

Binary outcome	Odds ratio (95% CI)	*p* value
Any compression versus normal	1.40 (1.23–1.64)	< 0.001
Absent versus visible	1.26 (1.15–1.41)	< 0.001

**Table 8:** Mean midline shift by pupil reactivity in diffuse lesions

Pupil responses	Mean MLS (mm)	Variance	*p* value
Normal versus unilaterally reactive versus bilaterally unreactive	0.15 versus 0.00 versus 0.33	0.81 versus 0.0 versus 0.67	0.22 (0.218 by JT)
At least unilaterally fixed versus other	0.14 versus 0.15	0.29 versus 0.81	0.48
Bilaterally fixed versus other	0.33 versus 0.13	0.67 versus 0.73	0.095

*JT* Jonckheere–Terpstra test, *MLS* midline shift

**Table 9:** Logistic regression results including age and basal cistern appearance in diffuse lesions

Predictor variable	Univariable regression odds ratio (95% CI; *p* value)		Multiple regression odds ratio (95% CI; *p* value)	
	Normal pupils versus at least unilaterally fixed	At least one pupil reactive versus bilaterally fixed	Normal pupils versus at least unilaterally fixed	At least one pupil reactive versus bilaterally fixed
Basal cisterns normal	Reference	Reference	Reference	Reference
Basal cisterns compressed	0.26 (0.065–1.04; 0.050)	0.55 (0.049–12.2; 0.63)	**0.22 (0.051**–**0.90; 0.033)**	0.52 (0.044–11.92; 0.61)
Basal cisterns absent	0.23 (0.047–1.33; 0.081)	**0.073 (0.0082**–**0.52; 0.0094)**	0.19 (0.033–1.13; 0.056)	**0.069 (0.0069**–**0.53; 0.011)**
Midline shift	1.01 (0.56–3.44, 0.986)	0.83(0.46–2.74; 0.589)	1.22 (0.66–4.66; 0.63)	1.03 (0.51–4.29; 0.94)
Age	0.99 (0.96–1.03; 0.55)	1.00 (0.95–1.06; 0.97)	0.98 (0.94–1.02; 0.33)	00.99 (0.94–1.06; 0.80)

Multiple regression includes basal cistern status, midline shift, and age as predictor variables. Bold *p* values are those reaching statistical significance (*p* < 0.05)

**Table 10:** Mean midline shift by basal cistern status in diffuse lesions

Basal cistern status	Mean MLS (mm)	Variance	*p* value
Normal versus compressed versus absent	0.00 versus 0.42 versus 0.59	0.00 versus 2.81 versus 1.47	0.0043 (0.009 by JT)
Normal versus any compression	0.00 versus 0.48	0.00 versus 2.25	0.0085
Visible versus absent	0.092 versus 0.59	0.61 versus 1.47	0.0022

*JT* Jonckheere–Terpstra test, *MLS* midline shift

**Table 11:** Univariable logistic regression results with midline shift as a predictor of basal cistern status in focal lesions

Binary outcome	Odds ratio (95% CI)	*p* value
Any compression versus normal	32.67 (0.0033–8702.04)^a^	0.92
Absent versus visible	1.47 (0.79–2.85)	0.17

^a^Given the small number of patients in the diffuse injury cohort, this value should be interpreted with caution

### Midline Shift as a Predictor of Pupil Reactivity

Logistic regression analysis was used to investigate the association between midline shift and the two dichotomizations of pupil reactivity. Using normal versus abnormal pupils as a binary outcome, midline shift trended toward statistical significance as a predictor of abnormal pupils (0.94; 95% CI 0.87–1.01; *p* = 0.077). When grouped by at least one reactive pupil versus bilaterally unreactive pupils, midline shift was not a significant predictor (0.98; 95% CI 0.89–1.12; *p* = 0.72). These relationships remained nonsignificant when pupillary status was weighted as bilaterally reactive = 0, unilaterally unreactive = 2, bilaterally unreactive = 4, in accordance with the IMPACT model [1, 3]. Midline shift also remained a nonsignificant predictor of pupillary reactivity when patients were grouped by focal versus diffuse injury (Tables 4, 5, 6, 7, 8, 9, 10, 11).

### Identifying a Midline Shift Threshold for Pupillary Abnormalities

**Fig. 2:**
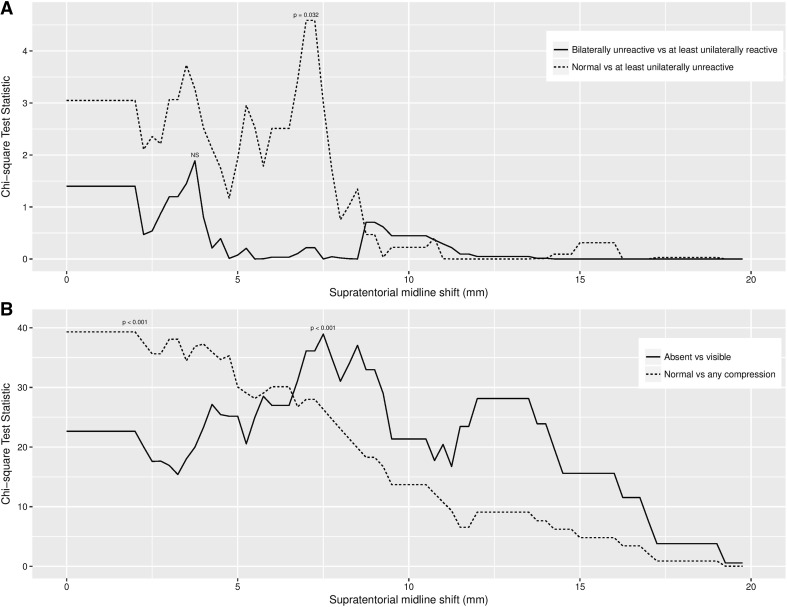
**a** Graph of Chi-square test statistic values for pupillary reactivity by midline shift threshold. **b** Graph of Chi-square test statistic values for basal cistern appearance by midline shift threshold. *NS* not significant

The Chi-square analysis for normal versus abnormal pupils demonstrated a significant peak at a midline shift threshold of 7 mm and 7.25 mm (Chi-square test statistic = 4.59; *p* = 0.032), though elsewhere there was no discernible distribution or statistically significant result. The Chi-square analysis for bilaterally unreactive versus other category failed to approach statistical significance at any point (Fig. 2).

### The Effect of Age

The effect of age was examined, with the hypothesis that the difference in ability to accommodate a mass lesion between younger and older patients would affect the relationship between midline shift and pupillary reactivity. Patients were dichotomized into two groups, age > 40 years and age ≤ 40, due to the increased tendency toward cortical atrophy at 40 [15]. When partitioned as such, there were no significant differences in mean midline shift for different pupillary reactivity categories. Partitioning into age groups did not improve the value of midline shift as a predictor of pupillary reactivity on univariate logistic regression analysis in either group.

Multiple regression analysis was used to model to the relationship between midline shift and pupillary reactivity while controlling for the effect of age. In the normal versus abnormal pupils group, the near-statistically significant effect of midline shift was lost. These results are presented with other regression analyses in Table 12.

**Table 12:** Logistic regression results including age and basal cistern appearance in the whole cohort

Predictor variable	Univariable regression odds ratio (95% CI; *p* value)		Multiple regression odds ratio (95% CI; *p* value)	
	Normal pupils versus at least unilaterally fixed	At least one pupil reactive versus bilaterally fixed	Normal pupils versus at least unilaterally fixed	At least one pupil reactive versus bilaterally fixed
Basal cisterns normal	Reference	Reference	Reference	Reference
Basal cisterns compressed	0.42 (0.17–1.02, *p* = 0.051)	**0.21 (0.041**–**0.89,** ***p* = 0.037)**	0.41 (0.16–1.02; *p* = 0.051)	**0.17 (0.033**–**0.74;** ***p*** **= 0.021)**
Basal cisterns absent	**0.21 (0.090**–**0.48,** ***p*** **< 0.001)**	**0.092 (0.020**–**0.33,** ***p*** **< 0.001)**	**0.18 (0.071**–**0.50;** ***p*** **< 0.001)**	**0.058 (0.01**–**0.23;** ***p*** **< 0.001)**
Midline shift	0.94 (0.87–1.01; *p* = 0.077)	0.98 (0.89–1.12; *p* = 0.72)	1.02 (0.93–1.12; *p* = 0.68)	1.11 (0.98–1.30; *p* = 0.14)
Age	0.99 (0.97–1.01; *p* = 0.41)	1.00 (0.97–1.03; *p* = 0.96)	0.99 (0.97–1.01; *p* = 0.38)	0.99 (0.96–1.02; *p* = 0.65)

Multiple regression includes basal cistern status, midline shift, and age as predictor variables. Bold *p* values are those reaching statistical significance (*p  *< 0.05)

### Midline Shift and Basal Cistern Status

Basal cisterns were graded via an ordinal system as either normal, compressed, or absent/completely effaced. Mean midline shift (mm) for patients in each category was 0.64, 2.97, and 5.93, respectively (*p* < 0.001). Applying the Jonckheere–Terpstra test, the sequential increase from normal to absent was statistically robust (*p* = 0.017). Mean midline shift was also significantly different in each binary categorization of basal cistern status (*p* < 0.001). Full results are available in Tables 13, 14. Logistic regression analysis revealed that midline shift predicts both basal cistern compression (OR 1.41; 95% CI 1.25–1.62; *p* < 0.001) and complete basal cistern effacement (OR 1.27; 95% CI 1.17–1.40; *p* < 0.001). All results remained significant on partitioning by age. When the population was partitioned by nature of injury, these relationships remained robust in patients with focal lesions, but midline shift was not a significant predictor of basal cistern compression in patients with diffuse injuries (Tables 4, 5, 6, 7, 8, 9, 10, 11).

**Table 13:** Mean midline shift by basal cistern status in the whole cohort

Basal cistern status	Mean MLS (mm)	Variance	*p* value
Normal versus compressed versus absent	0.64 versus 2.97 versus 5.93	3.32 versus 12.56 versus 40.13	< 0.001 (0.017 by JT)
Normal versus any compression	0.64 versus 4.38	3.33 versus 27.61	< 0.001
Visible versus absent	1.28 versus 5.93	6.90 versus 40.13	< 0.001

*JT* Jonckheere–Terpstra test, *MLS* midline shift

**Table 14:** Univariable logistic regression results with midline shift as a predictor of basal cistern status in the whole cohort

Binary outcome	Odds ratio (95% CI)	*p* value
Any compression versus normal	1.41 (1.25–1.62)	< 0.001
Absent versus visible	1.27 (1.17–1.40)	< 0.001

Sequential Chi-square analysis was applied to identify a threshold midline shift for basal cistern compression and complete basal cistern effacement. Statistically significant thresholds were identified as 2 mm for compression (Chi-square test statistic = 39.31, *p* < 0.001) and 7.5 mm for complete effacement (Chi-square test statistic = 38.94, *p* < 0.001) (Fig. 2).

### The Effect of Basal Cistern Status on Pupillary Reactivity

**Fig. 3:**
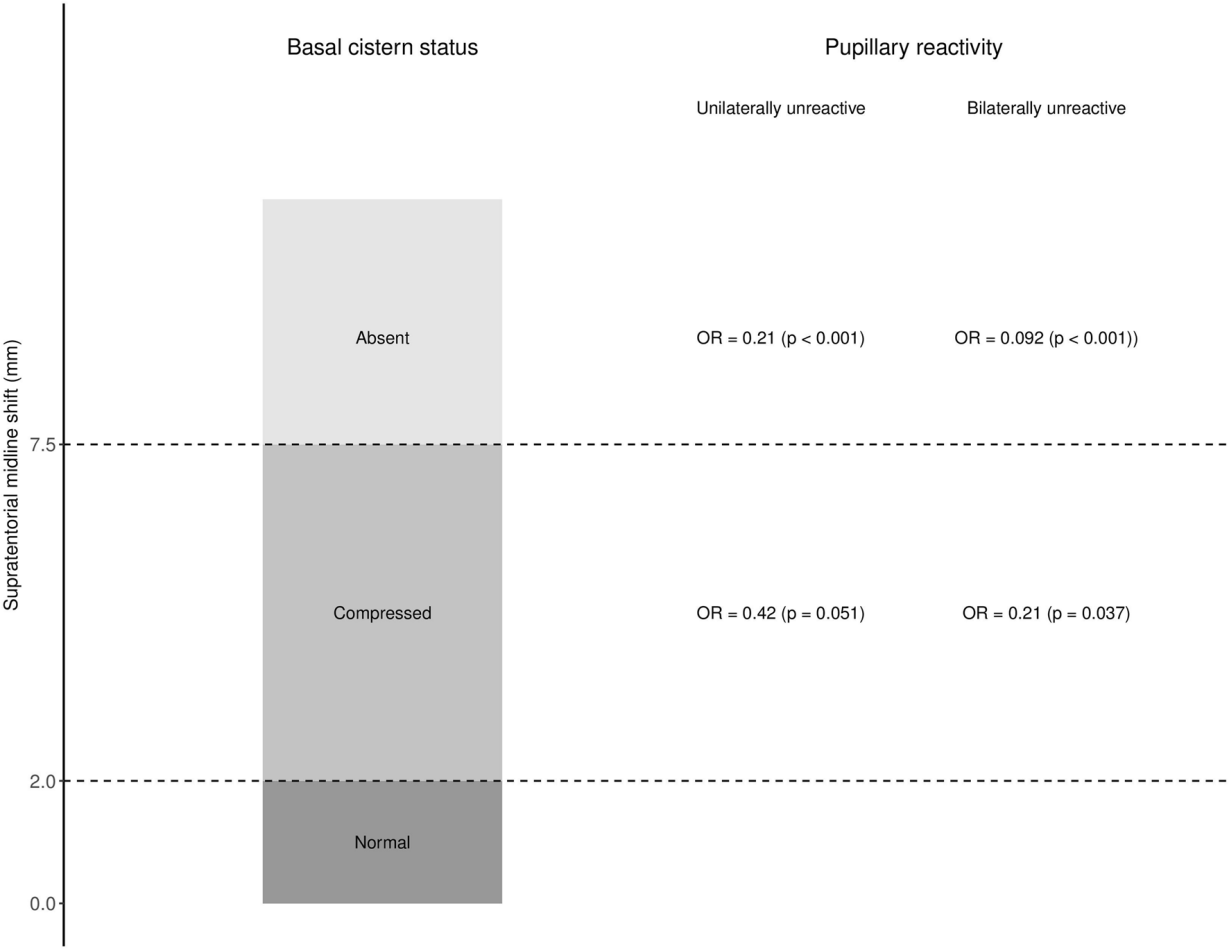
Basal cistern status by midline shift; odds ratio for normal pupillary reactivity by basal cistern status. *OR* odds ratio

Univariable logistic regression demonstrated a statistically significant predictive effect of complete basal cistern effacement for both normal versus abnormal pupils (OR 0.21; 95% CI 0.090–0.48; *p* < 0.001) and other versus bilaterally unreactive pupils (OR 0.092; 95% CI 0.020–0.33; *p* < 0.001) (Fig. 3). Multiple logistic regression analysis was performed including midline shift, age, and basal cistern status. In this model, basal cistern status was a statistically significant predictor both of normal versus abnormal pupils (OR with absent basal cisterns = 0.18; 95% CI 0.071–0.50; *p* < 0.001) and other versus bilaterally unreactive (OR with absent basal cisterns = 0.058; 95% CI 0.01–0.23; *p* < 0.001), while midline shift showed no statistically significant predictive value. These results are presented fully in Table 12. A significant relationship between basal cistern compression and abnormalities of pupillary reactivity is retained in both focal and diffuse lesions (Tables 4, 5, 6, 7, 8, 9, 10, 11). Finally, to further control for the effect of cortical atrophy affecting intracranial compliance and biasing results, the entire analysis was repeated excluding patients over the age of 50. Similar statistical relationships were observed (Tables 15, 16, 17, 18).

**Table 15:** Mean midline shift by pupil reactivity in patients ≤ 50

Pupil responses	Mean MLS (mm)	Variance	*p* value
Normal versus unilaterally reactive versus bilaterally unreactive	1.14 versus 2.25 versus 1.43	6.61 versus 16.43 versus 7.80	0.68 (0.19 by JT)
At least unilaterally fixed versus other	1.84 versus 1.14	12.13 versus 6.61	0.39
Bilaterally fixed versus other	1.43 versus 1.23	7.80 versus 7.49	0.641

*JT* Jonckheere–Terpstra test, *MLS* midline shift

**Table 16:** Logistic regression results including age and basal cistern appearance in patients ≤ 50

Predictor variable	Univariable regression odds ratio (95% CI; *p* value)		Multiple regression odds ratio (95% CI; *p* value)	
	Normal pupils versus at least unilaterally fixed	At least one pupil reactive versus bilaterally fixed	Normal pupils versus at least unilaterally fixed	At least one pupil reactive versus bilaterally fixed
Basal cisterns normal	Reference	Reference	Reference	Reference
Basal cisterns compressed	**0.24 (0.062**–**0.86; 0.027)**	0.32 (0.037–2.82; 0.27)	**0.23 (0.058**–**0.85; 0.026)**	0.28 (0.032–2.51; 0.23)
Basal cisterns absent	**0.098 (0.026**–**0.33; < 0.001)**	**0.069 (0.0095**–**0.33; 0.0019)**	**0.094 (0.024**–**0.34; < 0.001)**	**0.058 (0.0075**–**0.30; 0.0015)**
Midline shift	0.92 (0.80–1.09; 0.30)	0.98 (0.81–1.29; 0.82)	1.03 (0.87–1.24; 0.76)	1.11 (0.89–1.53; 0.43)
Age	1.02 (0.98–1.07; 0.38)	1.02 (0.97–1.10; 0.43)	1.01 (0.97–1.07; 0.56)	1.02 (0.95–1.09; 0.67)

Multiple regression includes basal cistern status, midline shift, and age as predictor variables. Bold *p* values are those reaching statistical significance (*p *< 0.05)

**Table 17:** Mean midline shift by basal cistern status in patients ≤ 50

Basal cistern status	Mean MLS (mm)	Variance	*p* value
Normal versus compressed versus absent	0.49 versus 2.10 versus 2.91	2.01 versus 12.47 versus 16.16	< 0.001 (0.001 by JT)
Normal versus any compression	0.49 versus 2.47	2.01 versus 13.99	< 0.001
Visible versus absent	0.90 versus 2.91	5.08 versus 16.16	0.0011

*JT* Jonckheere–Terpstra test, *MLS* midline shift

**Table 18:** Univariable logistic regression results with midline shift as a predictor of basal cistern status in patients ≤ 50

Binary outcome	Odds ratio (95% CI)	*p* value
Any compression versus normal	1.38 (1.17–1.69)	< 0.001
Absent versus visible	1.23 (1.07–1.43)	0.0047

## Discussion

This aim of this study was to characterize the relationship between pupillary reactivity and midline shift and basal cistern status on admission brain CT in TBI. Any mechanistic interaction between these factors is likely to relate to the effect of raised ICP due to supra-tentorial mass effect driving brainstem compression. The relationship between basal cistern effacement and raised ICP is well supported [16–19]. Similarly, past studies have generally—though not always [20, 21]—demonstrated an associative relationship between ICP and midline shift, though the extent of this relationship is likely to depend on the nature and location of the lesion, brain tissue compliance, and interhemispheric pressure gradients [16, 17, 22–24].

A direct relationship between pupillary reactivity and midline shift, an imaging marker of supra-tentorial mass effect, is not borne out in our data. A statistically significant difference between mean midline shifts among patients with different pupillary reactivity categories was found only for patients with at least one unreactive pupil compared to normal pupils. On logistic regression, midline shift fails to reach formal statistical significance for as a predictor of abnormal or bilaterally unreactive pupils, though trends toward it for the former. Inclusion of age and basal cistern appearance in multiple regression models causes midline shift to lose predictive significance entirely. As such, our results demonstrate no robust statistical evidence that midline shift is independently associated with pupillary reactivity. The results when extremes of age (> 50) were excluded were similar to the entire amalgamated cohort, though the impact of age is still unclear as this study is likely underpowered to detect meaningful differences between age groups.

Only basal cistern status, a direct imaging marker of 3rd cranial nerve/brainstem compression, appears to relate to a patient’s pupillary reactivity, both independently and in multiple regression models. However, basal cistern effacement also appears to be intimately linked with supra-tentorial midline shift, with mean midline shift increasing progressively for each ordinal category of cisternal effacement and logistic regression also demonstrating an increasingly significant relationship with compressed and absent basal cisterns, particularly when focal lesions are present. Interestingly, a similar midline shift threshold is identified for complete basal cistern compression (7.5 mm) and for the onset of abnormal pupillary reactivity (7–7.25 mm). This may reflect the late effect of progressive supra-tentorial mass effect driving transtentorial herniation, 3rd cranial nerve/brainstem compression and pupillary abnormalities, though the value of this specific threshold should not be overstated.

Significant midline shift (> 5 mm) is a key indication for surgical management in a number of traumatic brain lesions, including extra- and subdural hematomas and traumatic parenchymal lesions [25]. It typically results from unilateral frontal, parietal, or temporal lobe mass effect displacing the cingulate gyrus beneath the free edge of the falx cerebri and is associated with a number of neurological sequelae. Transtentorial herniation may coexist with or follow from subfalcine herniation, but may also occur in isolation. Uncal, parahippocampal, and central transtentorial herniation collective reflect a wider range of intercompartmental pressure effects and are all radiologically detected at an early stage by basal cistern compression [26]. As demonstrated in the present data, progressive basal cistern effacement on CT also has a close relationship with mesencephalic compression, a potentially disastrous complication of TBI. This emphasizes the value of basal cistern status, potentially over the traditional marker of midline shift, in guiding operative management in TBI. Further work is required to characterize imaging correlates of neurological deterioration in TBI, which will help to further develop robust indications for intervention among neurosurgeons and neurointensivists.

It is important to note that one would not expect basal cistern effacement to be absolutely predictive of pupillary reactivity. Altered pupillary reactivity in TBI is not exclusively a consequence of transtentorial herniation and mechanical compression of the oculomotor nerve and brainstem, with direct oculomotor nerve trauma [27] and brainstem ischemia [28] likely playing a role in some cases.

This study has a number of limitations, including its retrospective nature and relatively small patient numbers. The latter is particularly relevant to the sequential Chi-square analysis where the robustness of statistically significant results for thresholds must be interpreted with caution. The patient population is limited to patients admitted to NCCU who received multimodal monitoring, given these patients had archived available data. A further limitation is heterogeneity in treatment received during pre-hospital care and prior to CT scan acquisition. These treatments could have impacted both CT-based midline shift and basal cistern measurements and clinical pupillary reactivity. Notably, despite using admission data to minimize the risk of confounding by sedation, there is still a chance this occurred, as we did not have access to pharmacy records to exclude this. It is also important to note the potential impact of selection bias, as only 204 patients in our 358-patient database had accessible documented pupillary reactivity status on admission and the mean midline shift was significantly different in these two populations. Though there does exist the potential for variability in CT assessment of basal cisterns, as all scans were evaluated by a single consultant neurosurgeon, assessing interobserver variability in this regard was not the focus of this study.

 The scope of analysis was limited by the extent of recorded neurological examination. Pupil reactivity assessments were made subjectively by practitioners of varying experience and usually recorded as per our ordinal score without commenting on size or shape. While subjective pupil assessment is most widely used in clinical practice and therefore worthy of study, the use of automated assessment would provide a more reliable measure of pupillary reactivity. However, these data were not available in this retrospective cohort. Similarly, detailed brainstem reflexes were generally not assessed at the time of admission in the emergency department so no contemporaneous brainstem reflex data were available for analysis. Finally, it is unknown how the relationship between pupillary reactivity and imaging characteristics changed following medical or surgical intervention, as the only reliably contemporaneous data available for analysis were admission data. As such, we were unable to repeat the analysis after treatment. These are important questions for prospective study to further investigate the mechanisms, imaging correlates, and clinical significance of impaired pupillary reactivity.

## Conclusion

In this sample of moderate-to-severe TBI patients, supra-tentorial midline shift is not directly related to subjectively assessed pupillary reactivity. Basal cistern effacement is a robust predictor of impaired pupillary reactivity and is intimately associated with supra-tentorial midline shift. These results reinforce the idea that pupillary dysfunction is a late clinical correlate of progressive supra-tentorial mass effect and emphasize the utility of basal cistern effacement as a key neuroimaging characteristic to guide intervention in TBI.
